# Fourier transform infrared spectroscopy for the prediction of fatty acid profiles in *Mucor* fungi grown in media with different carbon sources

**DOI:** 10.1186/1475-2859-13-86

**Published:** 2014-09-11

**Authors:** Volha Shapaval, Nils Kristian Afseth, Gjermund Vogt, Achim Kohler

**Affiliations:** Nofima AS, Centre for Biospectroscopy and Data modeling, Osloveien 1, N-1430 Ås, Norway; Department of Mathematical Sciences and Technology, Norwegian University of Life Sciences, Postbox 5003, 1432 Ås, Norway

**Keywords:** FTIR spectroscopy, Microcultivation, Fungi

## Abstract

Fungal production of polyunsaturated fatty acids (PUFAs) is a highly potential approach in biotechnology. Currently the main focus is directed towards screening of hundreds strains in order to select of few potential ones. Thus, a reliable method for screening a high number of strains within a short period of time is needed. Here, we present a novel method for screening of PUFA-producing fungi by high-throughput microcultivation and FTIR spectroscopy. In the study selected *Mucor* fungi were grown in media with different carbon sources and fatty acid profiles were predicted on the basis of the obtained spectral data. FTIR spectra were calibrated against fatty acid analysis by GC-FD. The calibration models were cross-validated and correlation coefficients (R^2^) from 0.71 to 0.78 with RMSECV (root mean squared error) from 2.86% to 6.96% (percentage of total fat) were obtained. The FTIR results show a strong correlation to the results obtained by GC analysis, where high total contents of unsaturated fatty acids (both PUFA and MUFA) were achieved for *Mucor plumbeus* VI02019 cultivated in canola, olive and sunflower oil and *Mucor hiemalis* VI01993 cultivated in canola and olive oil.

**Electronic supplementary material:**

The online version of this article (doi:10.1186/1475-2859-13-86) contains supplementary material, which is available to authorized users.

## Introduction

The intake of polyunsaturated fatty acids (PUFAs) is important for human health and welfare. Some of the PUFAs are essential, meaning that they are not synthesized by the body; they must be supplied by food or other dietary sources. The main sources of PUFAs are fish oils, but also other sources for dietary PUFAs exist. Animal liver is an industrial source of arachidonic acid (ARA), some algae and mosses are good sources of eicosapentaenoic acid (EPA), and evening primrose oil is a source of γ-linolenic acid (GLA) [1]. All these sources have relatively low PUFA yield and high production costs. Thus, there is a need for alternative PUFA sources which are relatively inexpensive and which can produce the target fatty acids at high yields. Recent studies have shown that oleaginous fungi can serve as a possible source of PUFAs. Oleaginous fungi are metabolically versatile microorganisms which may produce from 48% to 66% of their dry weight as lipids [2, 3].

The most well-known oleaginous fungi originated from the division *Zygomycota* into several families where the families *Mortierellaceae* and *Mucoraceae* are of special concern. It has been shown that the commercially valuable gamma-linolenic acid (18:3 ω6) is produced by *Mucor* fungi [4], while arachidonic acid (ARA, 20:4 ω6), dihomo-gamma-linolenic acid (20:3 ω6) and mead acid (20:3 ω9) are produced by *Mortierella* fungi [5]. The important omega3 fatty acids eicosapentaenoic (EPA) and docosahexaenoic (DHA), which are mainly found in fish oils, can be produced by *Mortierella* and *Pythium* fungi and *Thraustochytrium* and *Schizochytrium* fungi, respectively [5, 6]. During recent years, several researchers have isolated and identified fungi producing PUFA [2].

In the search for possible PUFA producers, fungi are isolated and subsequently screened in different growth conditions. Often the isolated strains are genetically modified attempting to improve the PUFA content. Thus many different isolates, mutants and conditions need to be investigated in order to optimize the PUFA production. For this purpose the fatty acid profiles need to be analyzed. State of the art techniques for the determination of lipid profiles in microbial cells are gas chromatography and spectrophotometric methods such as fluorescence spectrometry. Also staining by Nile red for yeasts and algae, and staining by Nile blue and Luminor 490 T for oleaginous fungi [6] can be used for quantifying total lipid content. All of these techniques require the extraction of fats from the cells. Thus, they require complicated and time-consuming protocols limiting the number of conditions and microorganisms that can be screened.

Fourier transform-infrared (FT-IR) spectroscopy is a biophysical method that can be used for characterizing the biochemical composition of microbial cells. In microbial analysis in clinical medicine, food industry and basic research, FTIR spectroscopy is readily established as a routine analysis for rapid characterization and identification of microorganisms. Spectra are obtained from cells in their native, yet partially dehydrated, state. The acquired FTIR spectra represent a molecular ‘’fingerprint” of the total cell chemical composition. Thus, they constitute a unique fingerprint of cell lipids, proteins, nucleic acids and carbohydrates [7–9]. Since the phenotypic status of a microbial cell depends strongly on the cultivation conditions, it was early recognized that growth conditions have to be highly standardized when FTIR spectroscopy is used for identification of microorganisms in general, and particularly for fungi [8]. It was for example shown that FTIR spectroscopy can reveal small variations in cultivation parameters such as variations in culture time, medium composition and pH, temperature, water content or culture storage conditions [8, 10], [11]. Small changes in growth conditions may change the dynamics and products of metabolic pathways, leading to changes in the FTIR fingerprint. This demonstrates that FTIR can detect slight changes in the growth conditions demonstrating that it has a potential for monitoring the production of metabolic products in microbial fermentation.

The aim of this study was therefore to evaluate to what extent FTIR spectroscopy can be used to monitor the fatty acid profile of fungi depending on the type of strain and cultivation medium used. For this purpose five different fungal strains and five cultivation using three different oils (sunflower, canola or olive) and two different types of carbohydrates (glucose or sucrose), were used. In order to evaluate if FTIR spectroscopy can be used to estimate the fatty acid profile of fungi, multivariate calibration by partial least squares regression (PLSR) against Gas Chromatography (GC) analysis were applied. For the cultivation of fungi in different media, a recently developed system based on high-throughput liquid microcultivation and FTIR spectroscopy was used. The protocol was first developed for the identification and characterization of food spoilage moulds and yeasts where it showed a good reproducibility and sensitivity. The system was built for the characterization and identification of food spoilage fungi and yeasts, and can handle 200 samples in one run.

## Materials and methods

### Fungal strains

Five fungal strains from genus *Mucor* were used: *Mucor plumbeus* VI02019, *Mucor plumbeus* VI02022, *Mucor plumbeus* VI03754, *Mucor circinelloides* VI01914 and *Mucor hiemalis* VI01993. Fungi were obtained from the collection of the National Veterinary Institute (Oslo, Norway).

### Medium and culture conditions

All fungi were cultivated using 50 μl of spore inoculum and 300 μl of cultivation medium in an automated microcultivation Bioscreen C system (Oy Growth Curves AB, Helsinki, Finland). Spore inoculum of each fungal strain was prepared by collecting fungal spores from the malt extract agar plates with cotton tips followed by re-suspension in 10 mL of cultivation medium. Five cultivation media, containing 1% of yeast extract and 2% of either one of three oils (sunflower (Shodlik), canola (Odelia) or olive (Livio)) or 2% of one of two carbohydrates (glucose or sucrose), were used. Cultivation of fungi in the medium without oil was done as a control (malt extract broth). In order to have enough biomass for GC and FTIR analysis, each fungal strain was inoculated in 10 wells of a Bioscreen honeycomb plate. The cultivation was performed for 5 days at 25°C with continuous shaking.

### Preparation of mycelia for GC and FTIR spectroscopy analysis

Samples of the fungal mycelium were taken after 5 days of cultivation and prepared for GC and FTIR spectroscopy measurements. Each sample was prepared in the following way: (1) Mycelium was transferred from honeycomb plates to Eppendorf tubes, using a bacterial loop; (2) Mycelium was washed three times with distilled H_2_O and dried in a desiccator at room temperature for 36 hours; (3) Dried mycelium was sonicated with 2.5 mL of distilled water in a tip-sonicator for 30–60 seconds.

### FTIR spectroscopy analysis

Of each sonicated fungal suspension, 8 μl was transferred to an IR-light-transparent Silicon 384-well microtiter plate (Bruker Optik GmbH, Germany). The samples were dried at room temperature for 45 min to form films suitable for FTIR analysis. FTIR measurements were performed using a High Throughput Screening eXTension (HTS-XT) unit coupled to a Tensor 27 spectrometer (both Bruker Optik GmbH, Germany). The spectra were recorded in the region between 4000 and 500 cm^−1^ with a spectral resolution of 6 cm^−1^ and an aperture of 5.0 mm. For each spectrum, 64 scans were averaged.

### Lipids extraction for GC analysis

Fungal cell suspensions were sonicated and lipids were extracted from the sonicated suspensions. The extraction procedure had the following steps: (1) 2.5 mL of the sonicated fungal suspension was mixed with methanol, chloroform (volume ratio = 1:2:1). Phase separation was achieved by low speed centrifugation. The lower chloroform phase containing the lipids was removed and evaporated under N_2_. (2) Lipid samples were dissolved in benzene (1 mL/sample) and subsequently transmethylated with methanolic HCl (2 mL/sample) and 2,2-dimethoxypropane (200 mL/per sample) at room temperature overnight and afterwards at 80°C for 20 min [12]. The fatty acid methyl esters (FAME) were extracted with isooctane (1 mL/sample) and 5% NaCl (1 mL/sample), where NaCl was used for the purification of the solution by salting it out. Phase separation was achieved by low speed centrifugation for 1 min, the upper isooctane phase was removed and mixed with 2% NaHCO_3_ (1 mL/sample) and after phase separation, the upper phase was treated with water free Na_2_SO_4_.

### GC analysis

Fatty acid methyl esters (FAME) were estimated using GLC (6890 N, Agilent Technologies, Palo Alto, CA) with a split/splitless injector, an automatic liquid sampler (7683B), and flame ionization detection (FID). The presence of individual fatty acid was identified with standard samples (Larodan Fine Chemicals, Sweden), and their concentrations were calculated in percentage by weight of total fatty acid content. The major fatty acid features were expressed as summed fatty acid parameters: SAT (summed saturated fatty acids), MUFA (summed monounsaturated fatty acids) and PUFA (summed polyunsaturated fatty acids).

### Experimental design

All five fungi were subjected to all five cultivation media, resulting in 25 different samples (experimental conditions and strains). For each sample, three independent runs were performed at different days. In addition, for each run two parallel microcultivations of each sample were prepared in the Bioscreen C system. In each run, the 25 different samples were placed in 10 and 15 wells of the first and second Bioscreen micro-plate, respectively. For FTIR spectroscopy, three technical replicates were obtained from each Bioscreen micro-plate well, resulting in a total of 450 spectra. Subsequently, all spectra were subjected to a quality test (Bruker Analytik GmbH), according to which spectra with the following parameters were selected for further data analysis: (1) absorbance >0.35; (2) noise <1.5 × 10^−5^; (3) water vapor <3 × 10^−4^, few technical replicates which did not pass the quality test were removed from the data set.

### Data analysis

The infrared spectra were pre-processed in the following way. First, the number of spectra was reduced to 150 by taking averages of technical replicates. Then, second-derivative spectra were calculated using a nine-point Savitzky-Golay algorithm. Normalization was performed by applying extended multiplicative signal correction (EMSC) in the spectral region from 3050 cm-1 to 700 cm-1 [4]. The analysis of the pre-processed spectra was performed by partial least square regression (PLSR),which was used to find fundamental relations between two matrices X and Y, where FTIR data referred to as X- matrix and fatty acid reference values obtained by GC, referred to as Y-matrix, were used to develop multivariate regression models. Full cross-validation was applied to estimate the optimal number of PLSR factors of the calibration models. The optimal number of PLSR factors was estimated by testing if the root mean square error of cross-validation (RMSECV) changed significantly, when increasing the number of PLSR factors. For evaluation of the calibration models the RMSECV and the coefficient of determination (R^2^) between the reference and predicted values, were used. In addition, the 3200–2800 cm^−1^ IR region often referred to as the fatty acid region was analysed by principal component analysis (PCA).The basic principle of PCA is to transform the multivariate data from the original variables, which refer to wavenumbers in infrared spectroscopy, to new variables that maximize the variance in the data. Every new variable refers to a component defining a direction in the originally variable space: The first component refers to the direction representing maximum variance, the second component the direction representing the second most variance and so on. The PCA score plot was used to investigate the effect of carbon source on the lipid profile of fungi.

The Unscrambler, V10.01 (CAMO PROCESS AS, Oslo, Norway) and in-house developed algorithms in Matlab, V7.9 (The Mathworks, Inc., Natick, MA) were used to perform the analysis.

### Unsaturation index

The C = CH- vibration observed around 3010 cm^−1^ originates from unsaturated fatty acids inside the cells and can be used for examining the degree of unsaturation in lipids and oils. The integration limits for CH = CH: 3020–2993 cm^−1^, CH_2_: 2946–2902 cm^−1^.

The other important peak relevant for the lipid analysis occurs in the lower wavenumber region at 1740 cm^−1^ which assigned for C = O stretches of ester functional groups from lipid triglycerides and fatty acids, and therefore represents total lipids in the cell.

## Results and discussion

### The effect of carbon source on fatty acid profiles of *Mucor*strains

**Figure 1:**
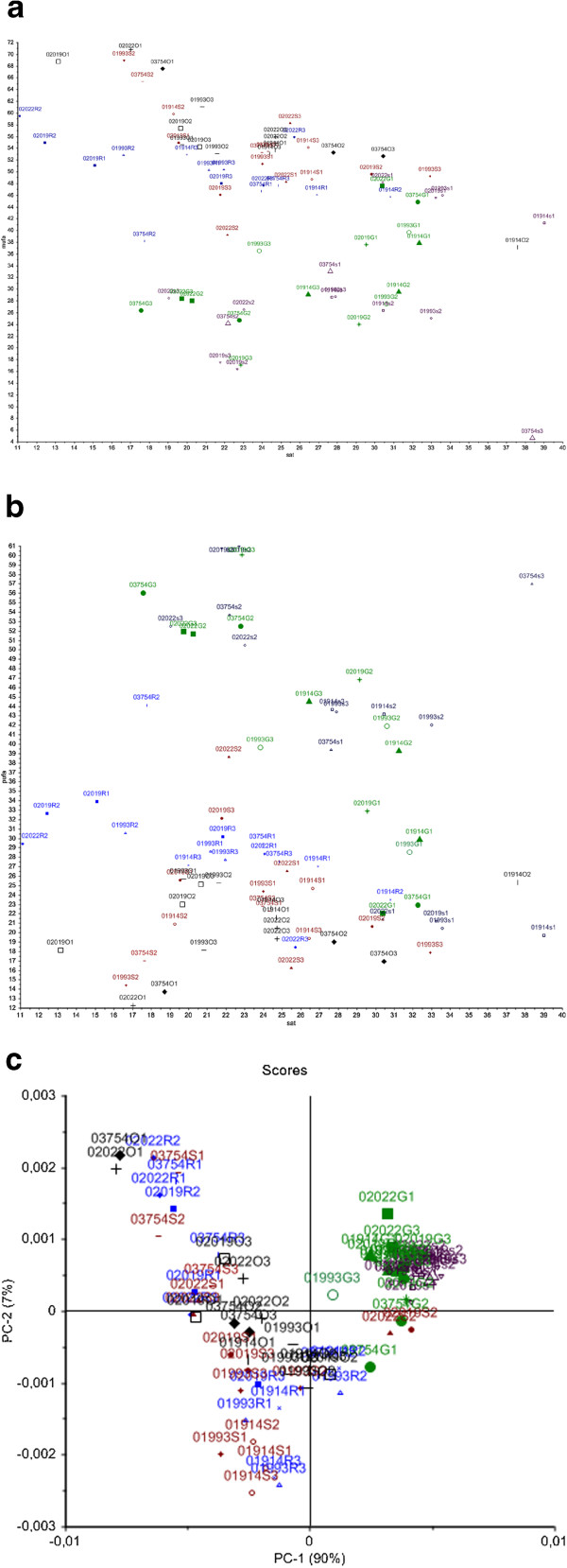
**Scatter and score plots of fatty acid parameters obtained by gas chromatography and FTIR spectroscopy. (a)**: Scatter plot of summed fatty acid parameters SAT versus MUFA of *Mucor* strains, obtained by gas chromatography. **(b)**: Scatter plot of summed fatty acid parameters SAT versus PUFA of *Mucor* strains, obtained by gas chromatography. **(c)**: Score plot of 3200–2800 cm^−1^ region of IR spectra of *Mucor* strains cultivated in the presence of carbohydrates.

Fatty acid results obtained by GC analysis of fungal mycelia are presented in Additional file 1: Tables S1 and S2, respectively. In Additional file 1: Table S1, results are presented for fungal cultivation using media containing 2% of sunflower, canola and olive oil, while in Additional file 1: Table S2 results are presented for fungal growth in media containing 2% of glucose and sucrose. Scatter plots of summed fatty acid parameters of the GC analysis of all *Mucor* strains are shown in Figure 1a and b, while a score plot of the principal component analysis (PCA) of the FTIR analysis of all *Mucor* strains is shown in Figure 1c. The summed saturated fatty acids (SAT) are plotted against the mono-unsaturated fatty acids in Figure 1a and the summed saturated fatty acids (SAT) are plotted against the poly-unsaturated fatty acids (PUFA) in Figure 1b. In Figure 1c a score plot of the PCA of the fatty acid region of the FTIR spectra (3200–2800 cm^−1^) is shown. It can be observed, that the most pronounced difference was obtained between cultivation media containing carbohydrate and oil containing sources (Figure 1a, b and c). The first principal component in Figure 1c accounting for 90% of the explained variation separated the fungi grown on media with carbohydrate sources and fungi grown on media with oil sources. Cultivation in media with oil sources resulted in a higher content of MUFA in fungal mycelium (Figure 1a), while the cultivation in media with carbohydrate source resulted in higher content of PUFA (Figure 1b).

**Figure 2:**
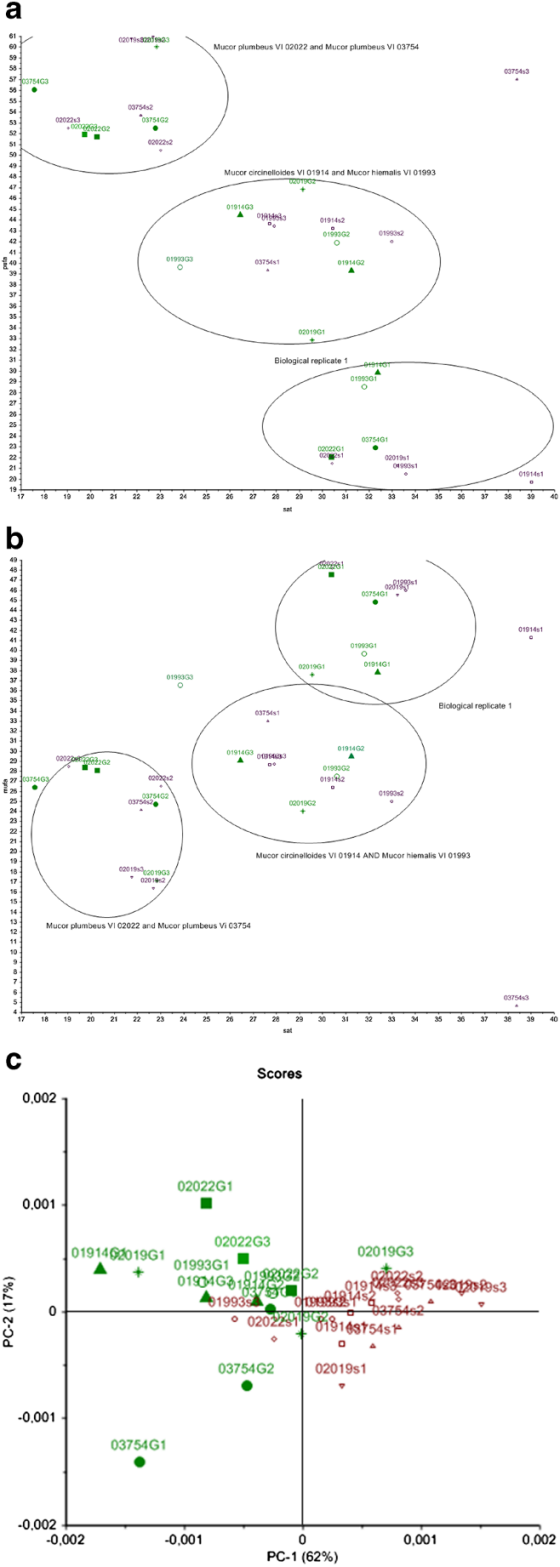
**Scatter and score plots of fatty acid parameters of** ***Mucor*** **strains cultivated in the presence of carbohydrates. (a)**: Scatter plot of summed fatty acid parameters of SAT versus PUFA of *Mucor* strains cultivated in the presence of carbohydrates. **(b)**: Scatter plot of summed fatty acid parameters of SAT versus MUFA of *Mucor* strains cultivated in the presence of carbohydrates. **(c)**: Score plot of 3200–2800 cm^−1^ region of IR spectra of *Mucor* strains cultivated in the presence of carbohydrates.

Scatter plots of the summed fatty acid parameters SAT versus PUFA and SAT versus MUFA and score plot of the PCA of the FTIR fatty acid region (3200–2800 cm^−1^) of *Mucor* strains, cultivated in carbohydrates source, is presented in Figure 2a, b and c, respectively. It can be seen that the strains *Mucor plumbeus* VI 02022 and *Mucor plumbeus* VI 03754, cultivated in both glucose and sucrose, showed high PUFA and low SAT (Figure 2a), while the strains *Mucor circinelloides* VI 01914 and *Mucor hiemalis* VI 01993 showed lower PUFA but higher SAT (Figure 2a). These four *Mucor* strains were clearly grouped in two separate clusters, indicating a similarity in the fatty acid profiles of *Mucor plumbeus* VI 02022 and *Mucor plumbeus* VI 03754, and *Mucor circinelloides* VI 01914 and *Mucor hiemalis* VI 01993 (Figure 2a and b). The content of MUFA showed less difference with respect to strain and carbon source (Figure 2b). Further it could be seen that strains analyzed in the first analytical run (biological replicate 1) formed their own cluster in the GC scatter plots (Figure 2a and b). This difference was obviously introduced during the preparation of samples for the GC analysis, since it cannot be observed in the score plot of the fatty acid region of the FTIR spectra (3200–2800 cm^−1^) (Figure 2c). Thus, the difference between biological replicates of the first run and the second and third run in the GC analysis is not connected to the microcultivation. While the difference between strains cultivated in glucose containing medium from strains cultivated in sucrose medium (Figure 2c) was clearly pronounced in FTIR data (Figure 2c), the same observation could not be made GC data. This may indicate a high variability in the obtained GC data, which was most likely the result of the sample preparation procedures used, while FTIR spectroscopy was more reproducible as sample preparation involved fewer steps.

**Figure 3:**
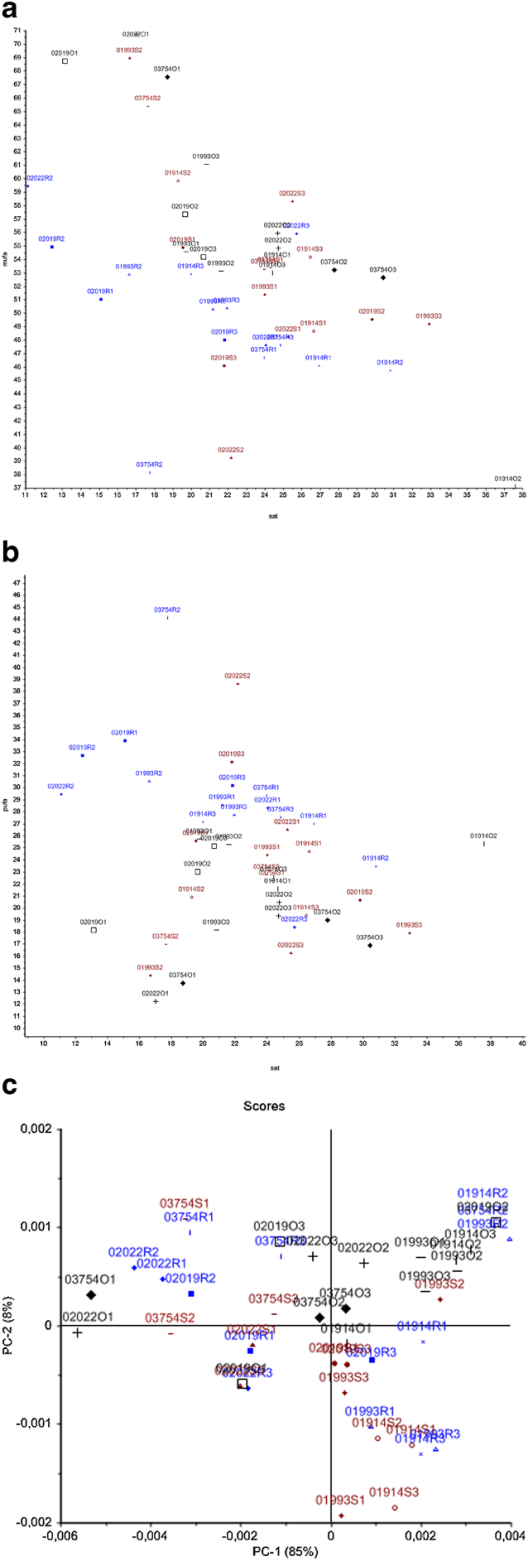
**Scatter and score plots of fatty acid parameters of** ***Mucor*** **strains cultivated in the presence of oils. (a)** Scatter plot of summed fatty acid parameters of SAT versus MUFA of *Mucor* strains cultivated in the presence of oils. **(b)** Scatter plot of summed fatty acid parameters of SAT versus PUFA of *Mucor* strains cultivated in the presence of oils. **(c)** Score plot of 3200–2800 cm^−1^ region of IR spectra of *Mucor* strains cultivated in the presence of oils.

In Figure 3a, b scatter plots of summed fatty acid parameters SAT versus PUFA and SAT versus MUFA are presented for *Mucor* strains cultivated in oil enriched media. The corresponding score plot of the PCA of the fatty acid region of the FTIR spectra (3200–2800 cm^−1^) is presented in Figure 3c. All three plots show a clear difference between strains cultivated in olive oil and canola oil. From Figure 3a and b it can be seen that the level of MUFA was higher after cultivation in olive oil, while the level of PUFA was higher after cultivation in canola oil. In addition a systematic difference in the PUFA production between strains cultivated in oil containing media and strains cultivated in carbohydrate containing media could be observed. The two strains *Mucor circinelloides* VI 01914 and *Mucor hiemalis* VI 01993, have in most cases a lower PUFA content than *Mucor plumbeus* VI 02022 and *Mucor plumbeus* VI 03754 strains. This difference is clearly detected in GC data after cultivation using a carbohydrate source (Figure 2a and b) and a bit less pronounced when oil sources were used. Similar to the GC analysis, the PCA analysis of the FTIR data separated the data clearly into two clusters: Cluster 1 containing *Mucor circinelloides* VI 01914 and *Mucor hiemalis* VI 01993 strains; and cluster 2 containing *Mucor plumbeus* VI 02022 and *Mucor plumbeus* VI 03754 strains. This systematic difference in the fatty acid profile between the two clusters of strains might indicate that the two groups of strains follow similar methabolic pathways related to the use of carbohydrate or fat substrates (Figure 2a, b and Figure 3a, b and c).

The observation of significantly different fatty acid profiles after cultivation in carbohydrate and oil enriched media may be due to the fact that fungi belonging to *Mucorales* generally are saprophytes which prefer to grow rapidly and proliferate extensively on simple sugars compared to complex molecules like oil. Carbohydrates are usually metabolized via the Embden-Meyerhoff pathway to generate pyruvate or acetyl-CoA. Thus, our results verify earlier observations showing that when carbohydrates are used as carbon sources PUFA formation is enhanced [1, 3, 5]. When in contrast oils are used as sources, fungi produce extracellular enzymes (i.e. lipases), which cleave fatty acid residues from glycerol, and the produced fatty acids are either incorporated in lipid structures or degraded to basic skeletons serving the biomass synthesis [5]. It has previously that when microorganisms are cultivated on animal and/or plant fat, little change usually occurs in the fatty acid profile of the accumulated lipids, when compared to the profile of the oil substrate [5]. This phenomenon is due to the fact that the desaturation and elongation processes are inhibited in the presence of substrate fatty acids [5].

### Analysis of the fatty acid profiles of *Mucor*strains by FTIR spectroscopy

**Figure 4:**
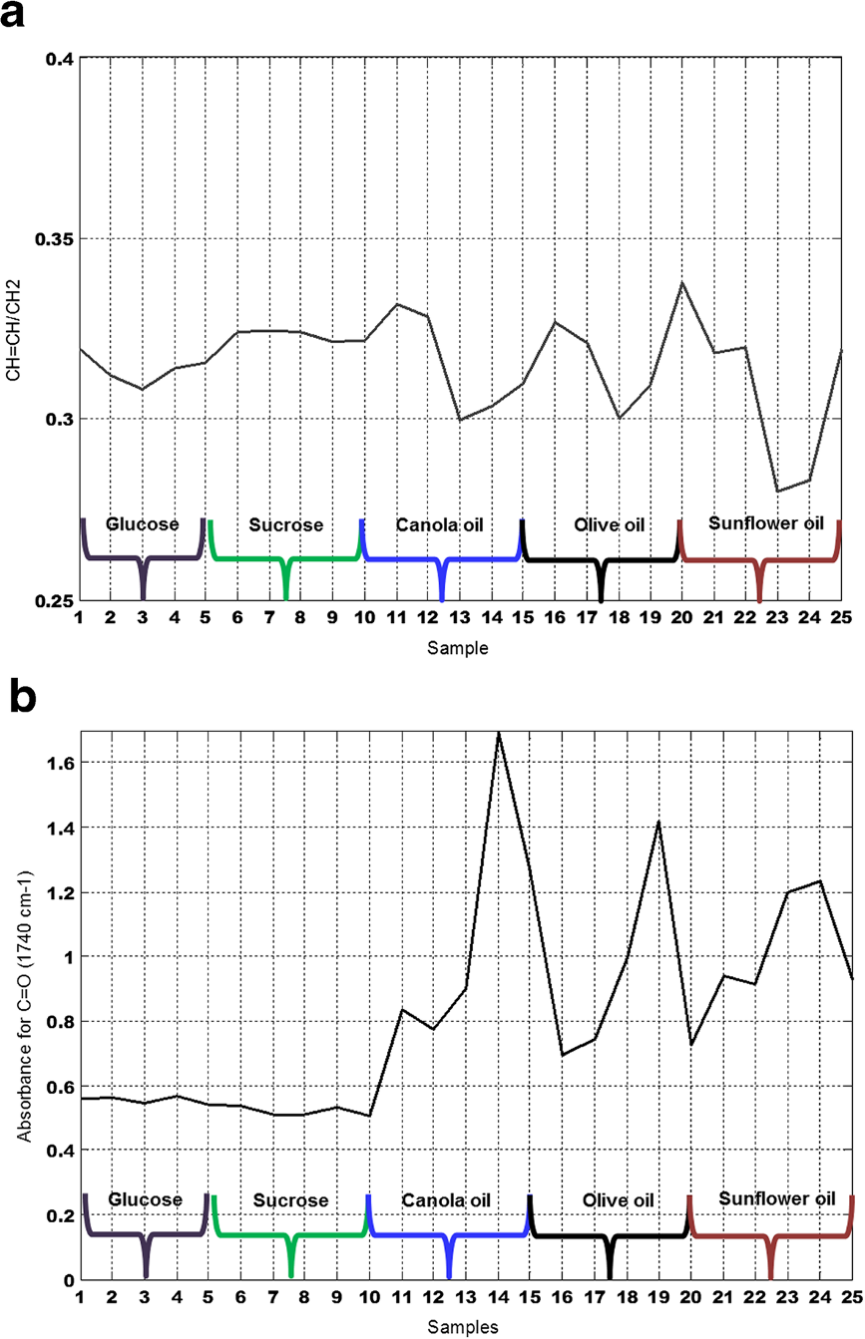
**Biomarkers of lipids in FTIR spectra. (a)** Ratio of peak area CH = CH (3020–2993 cm^−1^) versus CH_2_ (2946–2902 cm^−1^) of IR spectra of *Mucor* strains. **(b)** The absorbance at 1740 cm^−1^ (CH = O) of IR spectra of *Mucor* strains.

The spectral features in the range of 3100–2800 cm^−1^ of an FTIR spectrum are characteristic of the C-H stretching vibrations of lipids [12]. Thus, fatty acids characteristics obtained by FTIR spectroscopy may be presented in the form of FTIR bands ratios. An informative band ratio for the analysis of the fatty acid profile is the CH = CH/CH_2_ ratio. The ratio of the peak area CH = CH (3020–2993 cm^−1^) versus CH_2_ (2946–2902 cm^−1^) (henceforth denoted the unsaturation index) and the absorbance at 1740 cm^−1^ (assigned to the C = O stretch) of IR spectra of Mucor strains is presented on Figure 4a and b, respectively. Sample name is described in Table 1. Cultivation of *Mucor* strains in media enriched with carbohydrates and oils did not show a significant difference in unsaturation index with respect to carbon source, while the absorbance at 1740 cm^−1^ assigned for C = O stretches was much higher for strains cultivated in oil source than on carbohydrate source (Figure 4b). This indicates a higher lipid accumulation of fungal strains cultivated in oil. A slight difference in unsaturation index and absorbance at 1740 cm^−1^ was observed for strains cultivated in carbohydrate source, where values of unsaturation index were lower for all strains cultivated in glucose compared to the cultivation is sucrose (Figure 4a), while absorbance at 1740 cm^−1^ was slightly higher after cultivation in glucose source. The difference in values of the unsaturation index and the absorbance at 1740 cm^−1^ for strains cultivated in an oil source was strongly related to the strain type and less related to type of oil source. The high values of the unsaturation index and low values of absorbance at 1740 cm^−1^, meaning a high content of unsaturated fatty acids and low total lipid content, were observed for *Mucor plumbeus* VI02019 cultivated in olive and sunflower oil (sample #20 and #25), *Mucor hiemalis* VI01993 cultivated in canola and olive oil (sample #11 and #16) and *Mucor circinelloides* VI 01914 cultivated in canola, olive oil and sunflower oil (sample #12, #17 and #22), where *Mucor plumbeus* VI02019 cultivated in sunflower oil (sample #20) had the maximum value of unsaturation index, but lowest values of absorbance at 1740 cm^−1^ (Figure 4a and b). The low values of unsaturation index and high values of absorbance at 1740 cm^−1^, meaning low content of unsaturated fatty acids and high total lipid content, were observed for *Mucor plumbeus* VI 02022 cultivated in canola, olive and sunflower oil (sample #14, #19 and #24), *Mucor plumbeus* VI 02019 cultivated in canola oil (sample #15) and *Mucor plumbeus* VI 03754 cultivated in sunflower oil (sample #23) (Figure 4a and b).

**Table 1:** **Sample name and sample position in Figure** 4 **a and b**

Sample N.	Sample name	Cultivation medium
1	*Mucor hiemalis* VI 01993	2% sucrose
2	*Mucor circinelloides* VI 01914	2% sucrose
3	*Mucor plumbeus* VI 03754	2% sucrose
4	*Mucor plumbeus* VI 02022	2% sucrose
5	*Mucor plumbeus* VI 02019	2% sucrose
6	*Mucor hiemalis* VI 01993	2% glucose
7	*Mucor circinelloides* VI 01914	2% glucose
8	*Mucor plumbeus* VI 03754	2% glucose
9	*Mucor plumbeus* VI 02022	2% glucose
10	*Mucor plumbeus* VI 02019	2% glucose
11	*Mucor hiemalis* VI 01993	2% canola oil
12	*Mucor circinelloides* VI 01914	2% canola oil
13	*Mucor plumbeus* VI 03754	2% canola oil
14	*Mucor plumbeus* VI 02022	2% canola oil
15	*Mucor plumbeus* VI 02019	2% canola oil
16	*Mucor hiemalis* VI 01993	2% olive oil
17	*Mucor circinelloides* VI 01914	2% olive oil
18	*Mucor plumbeus* VI 03754	2% olive oil
19	*Mucor plumbeus* VI 02022	2% olive oil
20	*Mucor plumbeus* VI 02019	2% olive oil
21	*Mucor hiemalis* VI 01993	2% sunflower oil
22	*Mucor circinelloides* VI 01914	2% sunflower oil
23	*Mucor plumbeus* VI 03754	2% sunflower oil
24	*Mucor plumbeus* VI 02022	2% sunflower oil
25	*Mucor plumbeus* VI 02019	2% sunflower oil

The results obtained by FTIR spectroscopy have a strong correlation to the results obtained by GC analysis, where the high total content of unsaturated fatty acids (both PUFA and MUFA) that was achieved for *Mucor plumbeus* VI02019 cultivated in canola, olive and sunflower oil and for *Mucor hiemalis* VI01993 cultivated in canola and olive oil, was well correlated to the high values of unsaturation index and low values of absorbance at 1740 cm^−1^ obtained for these strains. The only exception was strain *Mucor plumbeus* VI02019 cultivated in canola oil showing the highest values of unsaturated fatty acids in the GC data but relatively low values of unsaturation index in the FTIR data.

There are four situations related to the content of unsaturated fatty acids versus total lipid content in the cell possible:

1. High content of unsaturated fatty acids and low total lipid content, indicated by *high unsaturation index versus low absorbance at 1740 cm*^*−1*^;
2. High content of unsaturated fatty acids and high total lipid content, indicated by *high unsaturation index versus high absorbance at 1740 cm*^*−1*^;
3. Low content of unsaturated fatty acid and high total lipid content, indicated by *low unsaturation index versus high absorbance at 1740 cm*^*−1*^;
4. Low content of unsaturated fatty acid and low total lipid content, indicated by *low unsaturation index versus low absorbance at 1740 cm*^*−1*^;

It is evident that the most attractive situations are situation 1 and 2, where content of polyunsaturated fatty acids is high. This may be due to attractive metabolic properties of the cells as in case of situation 1, where the PUFA content is high while the total lipid content is low, or due to the fact that the PUFA content is high since the total lipid content is high as in case of situation 2.

The results presented in this study show that when *Mucor plumbeus* VI02019 and *Mucor hiemalis* VI01993 are cultivated in sunflower oil, and when *Mucor circinelloides* VI 01914 and *Mucor hiemalis* VI01993 are cultivated in canola and olive oil, situation 1 is obtained.

**Figure 5:**
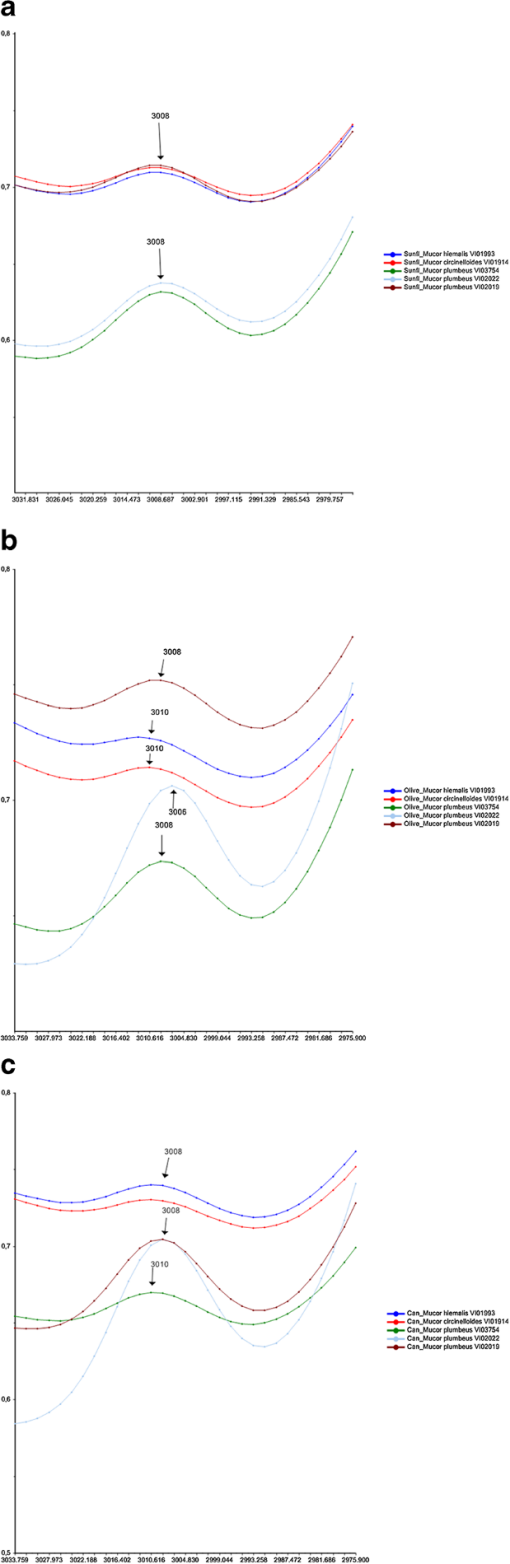
**Comparison of lipid content and composition for** ***Mucor*** **cultivated in different oils. (a)** Region 3030–2970 cm^−1^ of IR spectra of *Mucor* strains cultivated in sunflower oil source. **(b)** Region 3030–2970 cm^−1^ of IR spectra of *Mucor* strains cultivated in olive oil source. **(c)** Region 3030–2970 cm^−1^ of IR spectra of *Mucor* strains cultivated in canola oil source.

An FTIR index that can be used to study unsaturation is related to the FTIR band of the olefinic (CH = CH) double bond at 3020–2993 cm^−1^ (unsaturation index). Since the unsaturation index is also directly related to the hydrocarbon chain length [13], it does not provide a complete picture of the unsaturation level. It has also been shown that a shift of the band at 3008 cm^−1^ related to olefinic (CH = CH) double bonds reflect the degree of unsaturation: a shift of this peak maximum to lower wavenumbers suggests a higher degree of saturation [14, 15]. The FTIR region 3030–2870 cm^−1^ around the olefinic (CH = CH) double bond is shown for FTIR spectra of *Mucor* strains cultivated in sunflower, olive and canola oil in Figure 5a, b and c, respectively. A shift of the peak maximum for olefinic (CH = CH) double bonds from 3008 cm^−1^ to 3006 cm^−1^ is observed for *Mucor plumbeus* VI02022 cultivated in olive oil (Figure 5b), while in all other cases no shift was observed (Figure 5a, c). This is in accordance with the observed results for the unsaturation/acyl chain index (Figure 4a). The high degree of saturation observed in Figure 5b can be explained by the high content of saturated fatty acids in olive oil, which seem to be accumulated by *Mucor plumbeus* VI02022. The high absorption at 3008 cm^−1^/3006 cm^−1^ for *Mucor plumbeus* VI02022 might be an indication of high content of unsaturated fatty acids. Another relatively high absorbance values at 3008 cm^−1^ (olive and sunflower oil) and 3010 cm^−1^ (canola oil) was observed for strain *Mucor plumbeus* VI03754 (Figure 5a, b and c). An interesting observation was made in spectra of the strains *Mucor circinelloides* VI01914 and *Mucor hiemalis* VI01993 showing a shift from 3008 cm^−1^ to 3010 cm^−1^ after cultivation in olive oil: Although the absorption at the FTIR band related to the olefinic (CH = CH) double bond is low, the shift shows an increase in unsaturation and possibly a conversion of mono unsaturated/saturated toward more unsaturated fatty acids.

### Utilization of fat substrate: accumulation/conversion

The results obtained by FTIR spectroscopy illustrate that the composition of the fat substrate has a strong effect on fatty acid profile of fungal cells. The highest contents of unsaturated fatty acids for most of the fungal strains used in the study could be achieved after cultivation in canola oil and the lowest contents of unsaturated fatty acids were achieved by cultivation in olive oil. The fatty acid content of the fungi is correlated to the fatty acid composition of oils, where canola oil is highly unsaturated oil while olive oil contains approximately 14% of saturated fatty acids. Among all five fungal strains used in the study, a special attention should be given to the strain *Mucor plumbeus* VI02022 cultivated in canola oil (sample #20) which shows the highest unsaturation index with a low value of absorbance at 1740 cm^−1^ and high absorbance at 3008 cm^−1^, indicating a high content of unsaturated fatty acids after cultivation in canola oil. In addition, a high value of the unsaturation index with relatively low amount of total fat was obtained after cultivation of the same strain in olive oil. This may indicate an enzymatic accumulation of fatty acids when *Mucor plumbeus* VI02022 are cultivated on media containing Canola oils, a metabolic conversion of mono unsaturated and saturated fatty acids into more unsaturated fatty acids, when *Mucor plumbeus* VI02022 are cultivated on media containing olive oils. The analysis of peak shifts in FTIR spectra that are related to saturation showed that two other fungal strains (*Mucor circinelloides* VI01914 and *Mucor hiemalis* VI01993) might have an ability to convert mono unsaturated/saturated fat into unsaturated fatty acids.

### Prediction of fatty acids profile by FTIR spectroscopy

Partial least square regression models were developed for each summed fatty acid parameter versus reference values obtained by GC analysis. Results are presented in Table 2. In order to evaluate the calibration models the determination coefficients R^2^, root mean squared error of cross validation (RMSECV) and the number of PLSR factors used are shown. By applying 4 PLS factors, a R^2^ of 0.71 was achieved for summed saturated fatty acids (SAT). A 5 PLS factors PLSR prediction model resulted inR^2^ of 0.78 and 0.72 for the summed monounsaturated (MUFA) and polyunsaturated (PUFA) fatty acids, respectively. The RMSECV were 2.86%, 6.36% and 6.96% (percentage of total fat) for SAT, MUFA and PUFA, respectively. The obtained cross-validated errors are thus considered acceptable for monitoring the fatty acid profile in fungal cells. Based on the FTIR results discussed earlier in this study, it is also expected that the predictive models will improve further if a larger span in the variation of fatty acid composition is included in the calibration model.

**Table 2:** **Calibration results for prediction of fatty acids in** ***Mucor*** **fungi (N samples = 150)**

Fatty acid	RMSECV	R ^2^	Factors
SAT	2,86	0,71	4
MUFA	6,36	0,78	5
PUFA	6,96	0,72	5

## Conclusions

On-going research [6, 16–18] related to production of fungal oils is mainly directed towards a screening and isolation of fungal species producing target fatty acids with novel PUFA-related properties [19, 20] and the testing of different substrates and culture conditions to obtain high yield production of PUFAs [1, 21–24]. These are comprehensive research tasks, since they involve screening of hundreds of strains and testing of many different substrates and conditions, in order to find the best PUFA producers. The most crucial and time-consuming step in the screening is the quantification of fungal lipids. In the present study a high-throughput microcultivation protocol for FTIR spectroscopic analysis was successfully applied. The high-throughput microcultivation and preparation of mycelia for FTIR spectroscopic analysis presented in this paper can be completely performed by robotics and by that increase reproducibility and decrease technical variation.

## Electronic supplementary material

Additional file 1: Table S1: Mean of summed fatty acid parameters for fungal mycelia, cultivated in the presence of 2% of sunflower, canola and olive oil and in standard medium. Data were obtained from GC analysis. **Table S2.** Mean of summed fatty acid parameters for fungal mycelia, cultivated in the presence of 2% of sucrose and glucose. Data were obtained from GC analysis. (ODT 18 KB)
