# Supplementary material for: Fourier transform infrared spectroscopy for the prediction of fatty acid profiles in Mucor fungi grown in media with different carbon sources
Source: Microb Cell Fact. 2014 Sep 11;13:86. doi: 10.1186/1475-2859-13-86 (PMC4283129; doi:10.1186/1475-2859-13-86)
Supplement: Supplementary file 1 [file 12934_2013_1051_MOESM1_ESM.odt]

Table S1:  Mean of summed fatty acid parameters for fungal mycelia, cultivated in the presence of 2 % of sunflower, canola and olive oil and in standard medium. Data were obtained from GC analysis.

| Summed fatty acid     parameters | SAT1 | MUFA 1 | PUFA1 | Unsaturated fatty acids (TOTAL) |
| --- | --- | --- | --- | --- |
| Sunflo wer oil | Mucor plumbeus VI02019 | 20,9 | 45,7 | 33,2 | 78,9 |
| Mucor plumbeus VI02022 | 24,3 | 48,5 | 27,0 | 75,5 |
| Mucor plumbeus VI03754 | 21,9 | 57,3 | 20,6 | 77,9 |
| Mucor circinelloides VI01914 | 24,1 | 54,2 | 21,6 | 75,8 |
| Mucor hiemalis VI01993 | 24,4 | 55,9 | 19,5 | 75,4 |
| Canola oil | Mucor plumbeus VI02019 | 16,1 | 50,5 | 33,2 | 83,7 |
| Mucor plumbeus VI02022 | 20,3 | 54,3 | 25,3 | 79,6 |
| Mucor plumbeus VI03754 | 22,2 | 44,1 | 33,6 | 77,7 |
| Mucor circinelloides VI01914 | 25,9 | 48,2 | 25,8 | 74 |
| Mucor hiemalis VI01993 | 19,9 | 51,1 | 28,9 | 80 |
| Olive oil | Mucor plumbeus VI02019 | 16,7 | 58,9 | 24,2 | 83,1 |
| Mucor plumbeus VI02022 | 22,0 | 60,7 | 17,1 | 77,8 |
| Mucor plumbeus VI03754 | 25,1 | 57,1 | 17,6 | 74,7 |
| Mucor circinelloides VI01914 | 28,7 | 48,2 | 22,9 | 71,1 |
| Mucor hiemalis VI01993 | 20,6 | 56,1 | 23,2 | 79,3 |

 1 Range of fatty acid parameters (SAT – summed saturated fatty acids, MUFA – summed monounsaturated fatty acids, PUFA – summed polyunsaturated fatty acids) expressed as percentage of by weight of total acid content. 

Table S2:  Mean of summed fatty acid parameters for fungal mycelia, cultivated in the presence of 2 % of sucrose and glucose. Data were obtained from GC analysis.

| Summed fatty acid     parameters | SAT1 | MUFA 1 | PUFA1 | Unsaturated fatty acids (TOTAL) |
| --- | --- | --- | --- | --- |
| Sucrose | Mucor plumbeus VI02019 | 25,9 | 26,4 | 47,6 | 74 |
| Mucor plumbeus VI02022 | 24,1 | 34,3 | 41,4 | 75,7 |
| Mucor plumbeus VI03754 | 30,1 | 23,2 | 46,5 | 69,7 |
| Mucor circinelloides VI01914 | 32,3 | 32,1 | 35,5 | 67,6 |
| Mucor hiemalis VI01993 | 31,5 | 33,2 | 35,2 | 68,4 |
| Glucose | Mucor plumbeus VI02019 | 22,3 | 22,2 | 38,7 | 60,9 |
| Mucor plumbeus VI02022 | 23,4 | 34,6 | 41,8 | 76,4 |
| Mucor plumbeus VI03754 | 20,4 | 27,8 | 35,0 | 62,8 |
| Mucor circinelloides VI01914 | 30,0 | 32,1 | 37,8 | 69,9 |
| Mucor hiemalis VI01993 | 30,0 | 32,7 | 36,9 | 69,6 |

 1 Range of fatty acid parameters (SAT – summed saturated fatty acids, MUFA – summed monounsaturated fatty acids, PUFA – summed polyunsaturated fatty acids) expressed as percentage of by weight of total acid content.
